# Contribution of alternative splicing to breast cancer metastasis

**DOI:** 10.20517/2394-4722.2018.96

**Published:** 2019-03-22

**Authors:** Xiangbing Meng, Shujie Yang, Jun Zhang, Huimin Yu

**Affiliations:** 1 Department of Obstetrics and Gynecology, University of Iowa Carver College of Medicine, Iowa City, IA 52242, USA.; 2 Holden Comprehensive Cancer Center, University of Iowa Carver College of Medicine, Iowa City, IA 52242, USA.; 3 Division of Hematology, Oncology and Blood & Marrow Transplantation, Department of Internal Medicine, University of Iowa Carver College of Medicine, Iowa City, IA 52242, USA.; 4 Department of Pathogenic Biology, Shenzhen University School of medicine, Shenzhen 518060, China.

**Keywords:** Breast cancer, metastasis, CD44, MTDH, splicing, epithelial-mesenchymal transition

## Abstract

Alternative splicing is a major contributor to transcriptome and proteome diversity in eukaryotes. Comparing to normal samples, about 30% more alternative splicing events were recently identified in 32 cancer types included in The Cancer Genome Atlas database. Some alternative splicing isoforms and their encoded proteins contribute to specific cancer hallmarks. In this review, we will discuss recent progress regarding the contributions of alternative splicing to breast cancer metastasis. We plan to dissect the role of MTDH, CD44 and their interaction with other mRNA splicing factors. We believe an in-depth understanding of the mechanism underlying the contribution of splicing to breast cancer metastasis will provide novel strategies to the management of breast cancer.

## INTRODUCTION

Breast cancer is the most common type of cancer among women. Despite emerging new treatments such as PARP inhibition and immune checkpoint blockade, it remains a major challenge^[1,2]^ and is the primary cause of cancer mortality in women. In the majority of cases, the death from breast cancer is not due to the primary tumor per se, but rather the result of metastasis to other organs in the body^[3]^. Metastasis is a multistep process involving stromal invasion, cell migration, intravasation, anoikis resistance, extravasation and subsequent implantation and proliferation in sites other than the primary location^[4]^. Although we have gained ample knowledge in this cellular process, an in-depth understanding at the molecular level remains to be deciphered.

Alternative splicing might be such a molecular mechanism that contributes to metastasis. It is a process whereby multiple functionally distinct transcripts are encoded from a single gene by the selective removal or retention of exons and/or introns from the maturing RNA. This process is highly regulated, involving trans-acting splicing factors and cis-acting regulatory motifs and so is susceptible to hereditary and somatic mutations. Alternative splicing is common in many eukaryote lineages. Using deep transcriptome sequencing of the human genome, over 95% of multi-exon genes were found capable of producing at least one alternatively spliced isoform^[5]^. Many single-gene studies have also characterized the role of alternative splicing in various cellular processes. Disruption or dysregulation of alternative splicing has also been associated with pathological states^[6,7]^. Maguire *et al*.^[8]^ demonstrated that spliceosomal mutations occur in a mutually exclusive manner in breast cancer and that distinct components of the spliceosome are targeted by somatic mutations in different types of breast cancer. The exact splicing pattern associated with a particular breast cancer type or stage still requires a broad characterization through molecular analysis of splicing isoforms in different patients. However, existing evidence strongly supports a pivotal role of alternative splicing in breast cancer biology and innovative tools are under development to use splicing events for diagnostic and therapeutic purposes^[9]^. Shapiro *et al*.^[10]^ observed an epithelial-mesenchymal transition (EMT)-associated global change in alternative splicing of a number of genes that are involved in functions crucial for EMT progression, such as cell adhesion, cell motility, and cytoskeletal remodeling. Several of the splicing changes discovered *in vitro* were also found to occur in a panel of breast cancer cell lines and *in vivo* in primary human breast cancer samples. Dysregulation of alternative splicing has been increasingly recognized in cancer-related pathways. It is thus critical to investigate the functional significance of splicing regulation in the context of cancer. This review will discuss some recent progresses about the alternative splicing regulators such as CD44, heterogeneous nuclear ribonucleoprotein M (hnRNPM), SND1 and MTDH *etc*. in breast cancer metastasis.

## SPLICING VARIANTS MAY ENHANCE EMT AND METASTASIS IN BREAST CANCER

Dorman *et al*.^[12]^ reported splicing defects in large-scale breast cancer sequencing studies. Nine hundred and eighty-eight splicing variants including exon skipping, leaky or cryptic splicing from 5,206 putative mutations were confirmed for splicing mutations in 442 Breast Cancer patients from The Cancer Genome Atlas dataset. These splicing variants were significantly increased in patients with lymph node metastasis, but not in lymph node-negative tumors. Silipo *et al*.^[13]^ reported that the expression profile changes of splicing factors including serine/arginine-rich splicing factor 1 (SRSF1), SRSF2, SRSF3, SRSF5, SRSF6 and SRSF10; the heterogeneous nuclear ribonucleoproteins (hnRNPs) including hnRNP A2/B1, hnRNPI, hnRNPA1 and hnRNP K; as well as eight RNA-binding proteins including HuR, Sam68, BRM5, FOX2, YB-1, PRMT6, SPF45 and PELP1 in breast cancer cells compared with normal cells, which are strongly associated with the alternative splicing pattern of many cancer-related genes despite the absence of mutations in genomic DNA. Inoue *et al*.^[14]^ reported aberrant splicing of CD44 gene in breast cancer, which promotes metastasis. The status of splicing factors and other splicing-related proteins in breast cancer are important to provide insights into the mechanisms that lead to breast cancer metastasis^[15]^. The correlation analysis of somatic variants with alternative splicing events confirmed known trans- associations with variants in SF3B1 and U2AF1, and additional trans-acting variants (e.g., TADA1, PPP2R1A). Tumors have up to 30% more alternative splicing events than normal samples. Many tumors have thousands of alternative splicing events that are not detectable in normal samples. On average, 930 exon-exon junctions (‘‘neojunctions’’) were identified in tumors not typically found in normal tissue included in the Genotype-Tissue Expression (GTEx) project^[15]^. CD44 is a cell surface protein with various isoforms that involves in motility, cell survival and proliferation and the formation of tumor microenvironment. Alternative splicing can produce various isoforms of CD44 with properties at different specific tissue^[16,17]^. The RNA-binding protein hnRNPM was found to promote breast cancer metastasis by activating the switch of alternative splicing during EMT.

## HNRNPM INCREASES CD44 ALTERNATIVE SPLICING TO ENHANCE BREAST CANCER METASTASIS

CD44 was identified as a key downstream target of hnRNPM by genome-wide deep sequencing analysis. hnRNPM is associated with increased standard form of CD44 (or CD44 standard, CD44s) in aggressive breast cancer patient specimens. Overexpressed hnRNPM competes with epithelial splicing regulatory protein 1 (ESRP1), and binds to the same cis-regulatory RNA elements of CD44 for the precisely control CD44s splice isoform switching during EMT^[18]^. ESRP1 is a splicing regulator to promote an epithelial splicing program and hnRNPM is a mesenchymal splicing regulator. Harvey *et al*.^[19]^ reported that hnRNPM and ESRP1 co-regulate a set of cassette exon events in EMT genes associated with cell migration and cytoskeletal reorganization. Competitive binding to these cis-elements by hnRNPM and ESRP1 to antagonize alternative splicing was proposed. The expression of hnRNPM is closely correlated with invasion and metastasis of tumor cells. hnRNPM expression was upregulated in breast cancer tissues. HnRNPM and CD44s expression are positively correlated in breast cancer tissues. Cancer stem cells marker ALDH1+ was found positively associated with overexpression of CD44s and hnRNPM. High hnRNPM is associated with higher levels of CD44s, shorter overall survival and higher rates of lymph node metastases in breast cancer patients^[20]^.

## MORC2-MUTANT M276I PROMOTES AN HNRNPM-MEDIATED CD44 ALTERNATIVE SPLICING TO ENHANCE BREAST CANCER METASTASIS

**Figure 1.**
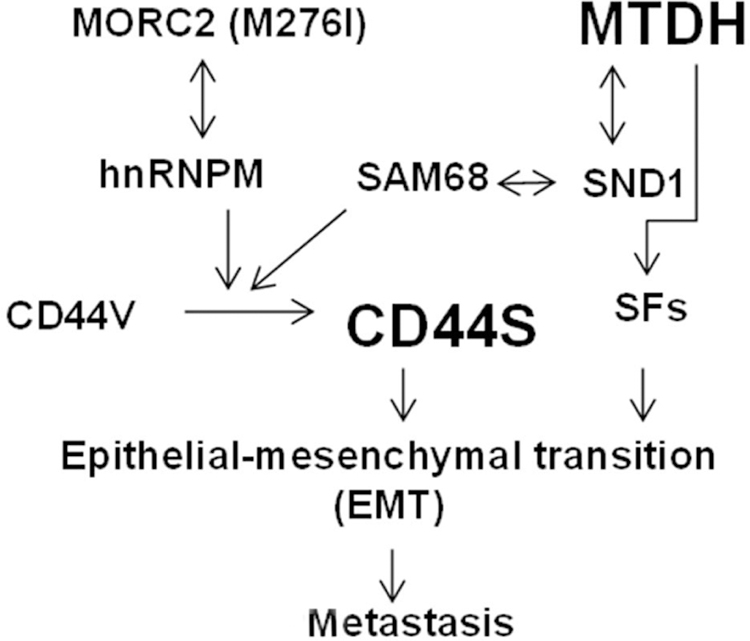
Mechanisms of alternative splicing of CD44 in epithelial-mesenchymal transition and breast cancer metastasis were summarized

A cancer-associated Microrchidia family CW-type zinc finger 2 (MORC2) (M276I) mutant was reported to promote metastatic ability of TNBC cancer cells by enhancing interaction with hnRNPM and splicing switch of CD44 from the epithelial isoform (CD44v) to the mesenchymal isoform (CD44s) [Figure 1]. Expression of mutant MORC2 in TNBC cells increased cell migration, invasion, and lung metastasis. The M276I mutation enhanced binding of MORC2 to hnRNPM, a component of the spliceosome machinery. This interaction promoted an hnRNPM-mediated splicing switch of CD44 from CD44v) to CD44s, ultimately driving EMT. Knockdown of hnRNPM reduced the binding of mutant MORC2 to CD44 pre-mRNA and reversed the mutant MORC2-induced CD44 splicing switch and EMT. As a consequence, the migratory, invasive, and lung metastatic potential of mutant MORC2-expressing cells was impaired^[21]^.

## TDP43, CPEB2A/B AND ESRP1/RBFOX2 IN BREAST CANCER METASTASIS

Ke *et al*.^[22]^ reported that the loss of TDP43 (TAR DNA-binding protein 43), an important splicing regulator involved in the SRSF3 regulated unique splicing of downstream gene PAR3, promotes metastasis in TNBC. Highly expressed TDP43 is correlated with poor prognosis in TNBC. Knockdown of TDP43 inhibits SRSF3 and PAR3 mediated metastasis. Two CPEB2 splicing isoforms with or without exon 4 was reported to mediate opposing effects on cancer-related phenotypes. The CPEB2A isoform, which is produced by exclusion of exon 4 from the mature CPEB2 mRNA, inhibited tumor growth. The CPEB2B splicing isoform with the inclusion of exon 4 into the mature CPEB2 mRNA was overexpressed in aggressive forms of human breast cancer and enhanced cancer metastasis was observed^[23]^. CPEB2A/B promotes the translation of two critical downstream proteins TWIST1 and HIF1a in the hypoxia/EMT pathway^[23]^. Splicing factor ratio might be an index of EMT and tumor aggressiveness in breast cancer. In fact, the association of low ESRP1/ RBFOX2 ratio with high risk of metastasis in early breast cancer was speculated to be a new early prognostic marker of breast cancer metastasis^[24]^.

## RNA BINDING PROTEIN RBM47 INHIBITS BREAST CANCER METASTASIS BY REGULATING SPLICING

RNA binding motif protein 47 (RBM47) was identified as a suppressor of breast cancer metastasis through analysis of clinical breast cancer gene expression datasets, cell line models, and mutation data. Transcriptome-wide HITS-CLIP analysis revealed widespread mRNAs associated with RBM47 by binding to their introns and 3’UTRs. The dickkopf WNT signaling pathway inhibitor 1 (DKK1) is one of downstream mRNAs of RBM47. RBM47 inhibits breast cancer metastasis by increasing stability of the Wnt antagonist DKK1^[25]^.

## NON-CODING RNA REST-003 PROMOTES BREAST CANCER METASTASIS

Non-coding RNAs (ncRNAs) RE1-silencing transcription factor (REST)-003 was reported to promote breast cancer metastasis. REST-003 is cRNAs derived from the first exon of an alternatively spliced REST transcript processed by serine/arginine repeat-related protein SRRM3. REST is a transcription factor to regulate expression of genes important for neuronal development. Interestingly, SRRM3 expression is repressed by REST^[26]^.

## MTDH PROMOTES BREAST CANCER METASTASIS PARTIALLY BY REGULATING ALTERNATIVE SPLICING

High metadherin gene expression was highly correlated with breast cancer metastasis^[27,28]^. MTDH was identified to bind to the vasculature of the lung by phage display screening cDNAs from metastatic breast carcinoma^[27]^. Experimental metastasis can be inhibited by metadherin specific antibody or siRNAs. Hu et al reported that MTDH drives breast cancer metastasis to the lungs by increasing adhesion to the walls of blood vessels^[28]^. Amplification of a minimal 2.9 Mb piece of chromosome 8q22 was identified in poor-prognosis breast cancers by ACE (analysis of CNAs by expression data) and fluorescence in situ hybridization (FISH) analysis. Only the enforced expression of MTDH in this amplified 8q22 region was identified to increase lung seeding after tail vein injection of the mildly metastatic breast cancer cell line MDA-MB-231^[28]^. Interaction of MTDH with Staphylococcal nuclease domain-containing 1 (SND1) was independently identified by mass spectrometry (MS) by three labs^[29–31]^. Overexpression of MTDH and SND1 in primary tumors is strongly associated with reduced metastasis-free survival in multiple large-scale datasets of breast cancer patients^[32,33]^. SND1 acts as a novel alternative splicing regulator by interacting with SAM68 to regulate exon v5 inclusion in the CD44 mRNA splicing that promotes cancer metastasis [Figure 1]^[34–36]^. Several other splicing regulators including hnRNPA0, hnRNPA2B1, hnRNPF, hnRNPA3 isoform were also identified by MS in MTDH pull-down assay^[29]^. High-throughput sequencing of RNA isolated by Cross-linking immunoprecipitation (HITS-CLIP) and Photoactivatable Ribonucleoside-Enhanced Cross-linking and Immunoprecipitation (PAR-CLIP) were recently developed methods to study RNA-protein interactions^[37,38]^. As shown in Table 1, mRNAs encoding for mRNA splicing regulators were identified in MTDH RNA interactome for multiple times at different sites by MTDH antibody specific PAR-CLIP and 12 splicing factors were confirmed by MTDH HITS-CLIP^[39]^. Therefore, MTDH may promote breast cancer metastasis by regulating mRNA splicing through interacting with mRNAs or proteins of splicing factors.

## CONCLUSION

Increased expression of mRNAs alternative splicing isoforms derived from alteration of splicing factors and MTDH expression could promote EMT and breast cancer metastasis, which provides new targets for breast cancer therapy.

## Figures and Tables

**Table 1.** List of mRNAs encoding for mRNA splicing factors identified by MTDH PAR-CLIP or HITS-CLIP

Ref Seq#	Gene	#PAR-CLIP	Ref Seq#	Gene	#PAR-CLIP
NM_006924	*SRSF1*	16	NM_004698	*PRPF3*	6
NM_003016	*SRSF2*	14	NM_014502	*PRPF19*	11
NM_003017	SRSF3*	7	NM_015629	*PRPF31*	3
NM_005626	*SRSF4*	8	NM_006109	*PRMT5*	7
NM_001039465	*SRSF5*	9	NM_014706	*SART3*	8
NM_006275	*SRSF6*	13	NM_018047	*RBM22*	5
NM_001031684	*SRSF7*	5	NM_012321	*LSM4*	5
NM_003769	*SRSF9*	7	NM_000344	*SMN1*	1
NM_054016	*SRSF10*	5	NM_015721	*GEMIN4*	11
NM_001190987	*SRSF11*	10	NM_015465	*GEMIN5*	4
NM_003090	*SNRPA1*	5	NM_024707	*GEMIN7*	3
NM_198216	SNRPB*	4	NM_024707	HNRNPA1*	7
NM_003093	*SNRPC*	5	NM_031157	HNRNPA2B1*	12
NM_006938	*SNRPD1*	3	NM_031243	HNRNPA3*	29
NM_004597	SNRPD2*	5	NM_194247	HNRNPH3*	10
NM_004175	*SNRPD3*	11	NM_012207	HNRNPK*	10
NM_003094	*SNRPE*	3	NM_002140	HNRNPL*	6
NM_003095	*SNRPF*	2	NM_001005335	HNRPU*	17
NM_003096	*SNRPG*	3	NM_004501	HNRNPUL2*	14
NM_022805	*SNRPN*	4	NM_001079559	PCBP1*	10

* MTDH interacting mRNAs identified in both High-throughput sequencing of RNA isolated by Cross-linking immunoprecipitation (HITS- CLIP) and Photoactivatable Ribonucleoside-Enhanced Cross-linking and Immunoprecipitation (PAR-CLIP)
